# Size scaling of photophysiology and growth in four freshly isolated diatom species from Ryder Bay, western Antarctic peninsula

**DOI:** 10.1111/jpy.12813

**Published:** 2019-01-29

**Authors:** Gemma Kulk, Anton Buist, Willem H. van de Poll, Patrick D. Rozema, Anita G. J. Buma

**Affiliations:** ^1^ Department of Ocean Ecosystems Energy and Sustainability Research Institute Groningen University of Groningen Nijenborgh 7 9747 AG Groningen the Netherlands; ^2^ Arctic Centre Faculty of Arts University of Groningen Aweg 30 9718 CW Groningen the Netherlands

**Keywords:** carbon uptake, diatoms, electron transport, photophysiology, size scaling, west Antarctic peninsula

## Abstract

Diatoms are one of the dominant groups in phytoplankton communities of the western Antarctic Peninsula (WAP). Although generally well‐studied, little is known about size dependent photophysiological responses in diatom bloom formation and succession. To increase this understanding, four Antarctic diatom species covering two orders of magnitude in cell size were isolated in northern Marguerite Bay (WAP). *Fragilariopsis* sp., *Pseudo‐nitzschia* cf. *subcurvata*,* Thalassiosira* cf. *antarctica*, and *Proboscia* cf. *alata* were acclimated to three different irradiances after which photophysiology, electron transport, carbon fixation, and growth were assessed. The small species *Fragilariopsis* sp., *Pseudo‐nitzschia* cf. *subcurvata*, and large species *Proboscia* cf. *alata* showed similar photoacclimation to higher irradiances with a decrease in cellular chlorophyll *a* and an increase in chlorophyll *a* specific absorption and xanthophyll cycle pigments and activity. In contrast, pigment concentrations and absorption remained unaffected by higher irradiances in the large species *Thalassiosira* cf. *antarctica*. Overall, the small species showed significantly higher growth rates compared to the large species, which was related to relatively high light harvesting capacity and electron transport rates in the smaller species. However, photophysiological responses related to photoinhibition and photoprotection and carbon fixation showed no relationship with cell size. This study supports the dominance of small diatoms at low irradiances during winter and early spring, but does not provide photophysiological evidence for the dominance of large diatoms during the phytoplankton bloom in the WAP. This suggests that other factors such as grazing and nutrient availability are likely to play a major role in diatom bloom formation.

**Abbreviations**
^14^C: carbon‐14
ā*: spectrally weighted mean specific absorption coefficient
C:Chl *a*: carbon to chlorophyll a ratio
Dd: diadinoxanthin
DPS: de‐epoxidation state of the xanthophyll pigment cycle
Dt: diatoxanthin
ETR: electron transport rate
ETR_max_: maximum electron transport rate
Eκ_,ETR_: photoacclimation index of electron transport
Eκ: photoacclimation index of carbon fixation
F_0_: minimum fluorescence in the dark‐adapted state
F_m_′: maximum fluorescence in the light
F_m_^o^: maximum fluorescence in the dark‐adapted state
F_m_^r^: maximum fluorescence in the absence of fast relaxing non‐photochemical quenching
F_t_: steady‐state fluorescence
Fuco: fucoxanthin
F_v_/F_m_: maximum quantum yield of photosystem II
NPQ_F_: fast non‐photochemical quenching
NPQ: non‐photochemical quenching
NPQ_S_: slowly non‐photochemical quenching
PAM: pulse amplitude modulation
PE: photosynthesis versus irradiance
P_max_: maximum carbon fixation rate
POC: particulate organic carbon
RaTS: rothera oceanographic and biological time series
S/V: surface‐to‐volume ratio
SSE: size scaling exponents
WAP: western Antarctic peninsula
α_ETR_: initial slope of electron transport
α: initial slope of carbon fixation
β‐car: β‐carotene
μ: growth rate
Φ_e,C_: electron requirement of carbon fixation
Φ_PSII_: quantum yield of photosystem II

Diatoms are one of the dominant taxonomic groups in coastal phytoplankton communities of the western Antarctic peninsula (WAP; Garibotti et al. 2003, Piquet et al. 2011, Rozema et al. 2017). The depth and geographic distribution of Antarctic diatoms and other taxonomic groups can be explained by their response to water column conditions (Garibotti et al. 2003, Piquet et al. 2011, Rozema et al. 2017). For example, diatoms thrive under shallow mixed water column conditions with relatively high, stable irradiance conditions, whereas haptophytes such as *Phaeocystis* spp. thrive under more deeply mixed conditions where short periods of darkness are interchanged with periods of excess irradiance at the surface (Arrigo et al. 1999, Kropuenske et al. 2009, Mills et al. 2010, Rozema et al. 2017). In the well‐studied northern Marguerite Bay, water column stability and the consequent irradiance climate phytoplankton experience are believed to control the onset of the phytoplankton bloom in spring (Clarke et al. 2008, Venables et al. 2013). The water column conditions in spring and summer are influenced by the preceding winter conditions, with the extent and duration of sea ice cover determining the strength of summer stratification and consequent mixed layer depth (Venables et al. 2013). Years with reduced sea ice cover are characterized by a deeply mixed water column in winter followed by a weaker stratified water column in summer and low biomass during the phytoplankton growth season (Meredith et al. 2010, Venables et al. 2013). Because diatoms play an important role in the Antarctic food web and carbon sequestering to the ocean's interior (Anadón and Estrada 2002, Ducklow et al. 2007), it is essential to understand how Antarctic diatoms respond to changes in water column conditions and how this influences the succession of the phytoplankton bloom.

Phytoplankton bloom dynamics in northern Marguerite Bay are well‐studied due to the presence of the Rothera Oceanographic and Biological Time Series (RaTS) station, located in Ryder Bay just off Adelaide Island (Clarke et al. 2008, Venables et al. 2013, Rozema et al. 2017). Although interannual variations exist, the phytoplankton bloom in Ryder Bay typically peaks in December and January with chlorophyll *a* concentrations reaching up to 20–25 mg · m^−3^ and is often followed by a second bloom in March (Clarke et al. 2008, Venables et al. 2013). The phytoplankton peak is dominated by larger microphytoplankton (>20 μm), whereas smaller pico‐ (<2 μm) and nanophytoplankton (2–20 μm) dominate prior and directly after the phytoplankton peak in October and March, respectively (Clarke et al. 2008, Montes‐Hugo et al. 2008). The winter phytoplankton community is dominated by nanophytoplankton when phytoplankton biomass is low (<0.02 mg Chl *a* · m^−3^; Clarke et al. 2008). Throughout the year, diatoms form the predominant taxonomic group in the phytoplankton community of northern Marguerite Bay, contributing over 90% of the total biomass during the bloom (Annett et al. 2010, Rozema et al. 2017). The dominance of specific diatom species varies throughout the season and among years, with high relative abundances of genera such as *Fragilariopsis*,* Minidiscus*,* Pseudo‐nitzschia*,* Thalassiosira*,* Odontella,* and *Proboscia* (Annett et al. 2010; A. Buma, pers. obs.).

Various laboratory studies have addressed physiological responses of individual Antarctic phytoplankton species to variations in water column conditions such as irradiance (Kropuenske et al. 2009, Arrigo et al. 2010, Mills et al. 2010), nutrients (Van de Poll et al. 2009, Alderkamp et al. 2012, Zhu et al. 2016), and elevated CO_2_ (Boelen et al. 2011, Trimborn et al. 2014, Hoppe et al. 2015). However, direct comparisons between different Antarctic phytoplankton species have often been limited to *Phaeocystis antarctica* and a single diatom species (Kropuenske et al. 2009, Arrigo et al. 2010, Alderkamp et al. 2012) and only a few studies have compared the diverse group of Antarctic diatoms in more detail (Karentz et al. 1991, Helbling et al. 1996, Timmermans et al. 2001a,b, Trimborn et al. 2013, 2014, Zhu et al. 2016). Antarctic diatoms are believed to dominate in shallow mixed layers with relatively high irradiance conditions due to effective photoprotection mechanisms, such as flexibility in photosystem II (PSII) connectivity, functional absorption cross section, de‐epoxidation of the xanthophyll pigment cycle and consequent non‐photochemical quenching (NPQ; Kropuenske et al. 2009, Van de Poll et al. 2011, Boelen et al. 2011, Trimborn et al. 2014). Moreover, Antarctic diatoms are able to photoacclimate to high irradiances by reducing cellular chlorophyll *a* and PSII reaction center abundance and/or antenna size and increasing cellular photoprotective pigmentation or other non‐photochemical processes (Kropuenske et al. 2009, 2010, Boelen et al. 2011). Typically, changes in light harvesting capacity are matched by an increase in the amount and/or activity of RUBISCO (Falkowski and La Roche 1991, MacIntyre et al. 1996), although this step is believed to be rate limiting in Antarctic diatoms (Young et al. 2014). Further research on Antarctic diatom species has revealed that smaller diatom species showed higher specific growth rates and thrive under lower irradiance and nutrient conditions compared to larger diatoms species (Karentz et al. 1991, Timmermans et al. 2001a,b). In temperate diatom species this has been attributed to a relatively high light harvesting capacity, higher susceptibility to photoinhibition, but higher nutrient uptake capacity and lower nutrient requirement in smaller compared to larger diatom species (Finkel 2001, Key et al. 2010, Grover 2011). This suggests that smaller diatom species would benefit from low irradiance and nutrient conditions, whereas larger diatoms species might exhibit more efficient photoprotection under the high irradiance conditions prevailing in shallow mixed layers, for example, caused by melt water stratification (Finkel 2001, Montes‐Hugo et al. 2008, Key et al. 2010).

Despite the importance of large diatom species such as *Proboscia* and *Thalassiosira* (cell length/diameter >150 μm and/or biovolume >6,000 μm^3^) during the phytoplankton bloom in Marguerite Bay (Annett et al. 2010, A. Buma, pers. obs.), experimental photophysiological research has mainly focused on smaller Antarctic diatom species such as *Fragilariopsis* and *Chaetoceros* (cell length <60 μm and/or biovolume <600 μm^3^; Timmermans et al. 2001a,b, Zhu et al. 2016). Since multiple studies have shown the importance of size scaling in phytoplankton performance (Raven 1998, Finkel 2001, Key et al. 2010), this study addressed the question whether size governs the photophysiological response of diatoms to irradiance conditions found in the coastal WAP. To this end, the following four Antarctic diatom species were freshly isolated in northern Marguerite Bay: *Fragilariopsis* sp., *Pseudo‐nitzschia* cf. *subcurvata*,* Thalassiosira* cf. *antarctica*, and *Proboscia* cf. *alata*. These co‐occurring diatoms differ in over two orders of magnitude in cell size and biovolume, as well as specific pigment fingerprint, and represent the diverse group of diatoms found in the WAP (Annett et al. 2010; A. Buma, pers. obs.). *Fragilariopsis* sp., *P. subcurvata*,* T. antarctica*, and *P. alata* were acclimated to three different irradiances and photophysiology, electron transport, carbon fixation and growth were assessed. Results are discussed in the context of size scaling and the influence of photoacclimation potential on the success of specific diatom species in natural phytoplankton communities in the coastal Antarctic waters of the WAP.

## Materials and Methods

### Isolation of Antarctic diatom species

The Antarctic diatom species *Fragilariopsis* sp., *Pseudo‐nitzschia* cf. *subcurvata*,* Thalassiosira* cf. *antarctica*, and *Proboscia* cf. *alata* were isolated at the long‐term RaTS station (67°34.20′ S, 68°13.50′ W) in northern Marguerite Bay, WAP in January‐February 2014. *Fragilariopsis* sp. and *P. subcurvata* were isolated using 10% serial dilutions in f/2 + Si medium based on natural oceanic seawater (Guillard 1975). *Thalassiosira antarctica* and *P. alata* were manually isolated using light microscopy (100–200× magnification, Zeiss Axiomat microscope) and sterilized Pasteur pipettes and post isolated using 10% serial dilutions in f/2 + Si medium. Species identification was performed using light microscopy according to Scott and Thomas (2005). Additional analysis of 18S rRNA sequences (MiSeq, Illumina), annotated using the PR^2^ database with curated taxonomy (Guillou et al. 2013), from environmental samples collected at RaTS showed the presence of identical genera (P.D. Rozema, unpub. data). The Antarctic diatom species were subsequently cultivated in f/2 + Si medium in 100 mL glass Erlenmeyer flasks at 10 μmol photons · m^−2^ · s^−1^ (Biolux lamps, Osram) in a diurnal cycle of 16:8 h light:dark at 4°C.

### Experimental design

Cultures of *Fragilariopsis* sp., *Pseudo‐nitzschia subcurvata*,* Thalassiosira antarctica*, and *Proboscia alata* were transferred to 500 mL glass Erlenmeyer flasks and incubated in triplicate (*n* = 3) at 10, 50, and 100 μmol photons · m^−2^ · s^−1^. The three experimental irradiances were provided as a square wave function with a 16:8 h light:dark cycle in a U‐shaped lamp setup (for details see Van de Poll et al. 2007). The irradiance levels in the setup were frequently monitored using a QSL‐2101 (Biospherical Instruments, Santa Clara, CA, USA). The temperature in the setup was maintained at 1°C by a thermostat (RK 8 KS, edition 2000; Lauda Dr. R. Wobser & Co., Lauda‐Königshofen, Germany) and deviated less than ±0.5°C. After an acclimation period of 14 d under the experimental irradiance and temperature conditions, cultures of the four diatom species were transferred to fresh f/2 + Si medium to start the experiment. During the experiment, growth and maximum quantum yield of PSII (F_v_/F_m_) were followed daily starting directly after the beginning of the incubation. In the mid‐exponential growth phase (t = 6–14 d), cellular characteristics of the cultures were assessed by the analysis of cell size, biovolume, and cellular carbon, photophysiology was assessed by the analysis of pigments, absorption spectra, F_v_/F_m_, and NPQ, and photosynthetic rates were assessed by the analysis of electron transport rates (ETR) and carbon fixation rates.

### Growth measurements

Samples for cell counts were collected daily during the exponential growth phase. Duplicate samples (2 mL) of each replicate culture were fixed using lugol (0.04% final concentration) and formaline (0.04% final concentration) and cell concentrations were determined by light microscopy (Standard WL; Zeiss, Oberkochen, Germany) according to LeGresley and McDermott (2010) using an improved Neubauer counting chamber for *Fragilariopsis* sp. and a Sedgewick‐Rafter counting chamber for *Pseudo‐nitzschia subcurvata*,* Thalassiosira antarctica* and *Proboscia alata*. Growth rates (μ · d^−1^) of the exponential growth phase were calculated by linear regression of natural log‐transformed cell numbers for all replicates (≥4 data points).

In addition to cell counts, cell dimensions were measured in samples collected in the mid‐exponential growth phase for each replicate culture using light microscopy (Standard WL; Zeiss). Following Hillebrand et al. (1999), cell dimensions (*n* = 75) were estimated by the measurement of the apical, transapical, and pervalvar axis (cell length, width, and height, respectively) for *Fragilariopsis* sp. and *Pseudo‐nitzschia subcurvata* and by the cell diameter and height for *Thalassiosira antarctica* and *Proboscia alata*. Measurements of cell dimensions were then used to calculate biovolume, cell surface, and surface‐to‐volume (S/V) ratios according to Hillebrand et al. (1999).

### Cellular carbon

Samples for particulate organic carbon (POC) analysis were taken during the mid‐exponential growth phase for each replicate culture. Duplicate samples (15–30 mL) were filtered onto precombusted (4 h, 600°C) 12 mm GF/F filters (Whatman, Maidstone, United Kingdom), snap frozen in liquid nitrogen, and stored at −80°C until further analysis. For analysis, filters were acidified under HCl (37%) fumes for 4 h, dried overnight at 60°C, and wrapped in tin capsules (Elemental Microanalysis Ltd., Okehampton, United Kingdom). Analysis of the samples was performed on a cavity ring‐down spectrometer type G2101‐I (Picarro, Santa Clara, CA, USA) with a combustion module (Costech, Santa Clara, CA, USA).

### Pigment composition

Samples for pigment analysis were taken during the mid‐exponential growth phase for each replicate culture. Samples (15–60 mL) were filtered onto 25 mm GF/F filters (Whatman), snap frozen in liquid nitrogen and stored at −80°C until further analysis. Pigments were quantified using high‐performance liquid chromatography (HPLC) as described by Van Heukelem and Thomas (2001) and modified according to Perl (2009). In short, filters were freeze‐dried for 48 h and pigments were immediately extracted in 3 mL 90% acetone (v/v, 48 h, 4°C). Detection of pigments was carried out using a HPLC (Waters 2695 separation module, 996 photodiode array detector) equipped with a Zorbax Eclipse extra dense bonding C_8_ 3.5 μm column (Agilent Technologies, Santa Clara, CA, USA). Peaks were identified by retention time and diode array spectroscopy. Pigments were quantified using standard dilutions (DHI LAB products) of chlorophyll *a* (Chl *a*), chlorophyll c_2_ (Chl‐c_2_) chlorophyll c_3_ (Chl‐c_3_), fucoxanthin (Fuco), diadinoxanthin (Dd), diatoxanthin (Dt), and β‐carotene (β‐car). The de‐epoxidation state (DPS) of the xanthophyll pigment cycle was calculated as Dt/(Dd + Dt).

### Absorption spectra

Samples for pigment absorption spectra were taken during the mid‐exponential growth phase for each replicate culture. Phytoplankton pigment absorption spectra were determined on a Cary 3E UV‐Vis spectrophotometer (Varian, CA, USA), equipped with an integrating sphere. Spectral values of the absorption coefficient were recorded every 1 nm between 300 and 800 nm. For analysis, 20–60 mL culture sample was filtered onto 25 mm GF/F filters (Whatman) and the transmission and reflection of the total particulate matter was determined according to Tassan and Ferrari (1995). The filter was then extracted in sodium hypochlorite (1% chlorine) to remove phytoplankton pigments and measured again to obtain the absorption of non‐pigmented material (detritus). Phytoplankton absorption was calculated and normalized to Chl *a* concentrations to obtain the specific absorption coefficient by phytoplankton a*_ph_(λ) (m^2^ · mg Chl *a* ^−1^). The irradiance used in the photosynthetron during carbon fixation measurements was used to obtain the spectrally weighted mean specific absorption coefficient ā* (m^2^ · μg Chl *a* ^−1^) between 400 and 700 nm.

### PSII chlorophyll fluorescence characteristics

PSII fluorescence analyses were performed on a pulse amplitude modulation (PAM) chlorophyll fluorometer (Waltz GmbH, Bad Waldsee, Germany) equipped with a WATER emitter‐detector (ED) unit and analyzed using WinControl software (version 2.08; Waltz GmbH) according to Maxwell and Johnson (2000) (and references therein) and Kulk et al. (2012, 2013). For daily analysis, 10 mL samples were dark adapted for 20 min at 1°C. Then, F_0_ was recorded as the minimal fluorescence and F_m_^o^ as the maximum fluorescence in the dark‐adapted state. F_v_/F_m_ was calculated as (F_m_^o^ − F_0_)/F_m_^o^.

In addition to the daily analysis of F_v_/F_m_, NPQ and ETR were assessed in the mid‐exponential growth phase. Both measurements were performed in a climate controlled room at 1°C that deviated less than ±0.5°C. For measurements of NPQ, 10 mL samples were dark adapted for 20 min at 1°C, after which the F_v_/F_m_ was recorded as described above. Samples were then exposed to high irradiance (456 μmol photons · m^−2^ · s^−1^ provided by a blue led at 460 nm) for 5 min after which the quantum yield of PSII (Φ_PSII_) was determined every 5 min during a recovery period of 1 h by measuring F_t_ as the steady‐state fluorescence prior to the saturating light flash and F_m_′ as the maximum fluorescence in the light. Φ_PSII_ was calculated as (F_m_′ − F_t_)/F_m_′. From the F_v_/F_m_ measurements at t = 0 min and the Φ_PSII_ measurements at t = 5 min, total NPQ was calculated as (F_m_^o^ − F_m_′)/F_m_′. Relaxation analysis was performed to calculate the contribution of slowly and fast relaxing NPQ to estimate photoinhibition and photoprotection, respectively. (Walters and Horton 1991, Osmond 1994, Maxwell and Johnson 2000). To this end, F_m_′ values were corrected for F_0_ and extrapolated to estimate the value of F_m_′ that would have been attained if only slowly relaxing quenching was present in the light (F_m_^r^). Slowly relaxing non‐photochemical quenching (NPQ_S_) was then calculated as (F_m_^o^ − F_m_^r^)/F_m_^r^ and fast relaxing non‐photochemical quenching (NPQ_F_) as (F_m_^o^/F_m_′) − (F_m_^o^ − F_m_^r^).

For measurements of ETR, duplicate 10 mL samples were dark adapted for 20 min at 1°C. The F_v_/F_m_ was recorded at 0 μmol photons · m^−2^ · s^−1^ and the Φ_PSII_ was recorded after 1 min exposure to 7 different irradiance levels ranging from 8 to 456 μmol photons · m^−2^ · s^−1^ provided by the actinic light of the WATER‐PAM (blue led, 460 nm). The absolute ETR (ETR in mol e · μg Chl *a* ^−1^ · h^−1^) for each irradiance level was calculated by Φ_PSII_ × *E* × ā* × 0.5, where *E* (μmol photons · m^−2^ · s^−1^) is the irradiance level of the WATER‐PAM, ā* (m^2^ · mg Chl *a* ^−1^) is the spectrally weighted mean specific absorption coefficient, and 0.5 is a factor accounting for the partitioning of energy between photosystem I (PSI) and PSII. ETR versus irradiance curves were fitted to the empirical model described by Platt et al. (1980) using MatLab software (version 8.3; Mathworks, Natick, MA, USA) to estimate the maximum electron transport rate (ETR_max_ in mol e · μg Chl *a* ^−1^ · h^−1^), the initial slope of electron transport (α_ETR_ in mol e · μg Chl *a* ^−1^ · h^−1^ (μmol photons · m^−2^ · s^−1^)^−1^), and the photoacclimation index of electron transport (Eκ_,ETR_ in μmol photons · m^−2^ · s^−1^).

### Carbon fixation

Samples for carbon fixation rates were taken during the mid‐exponential growth phase for each replicate culture. A ^14^C‐bicarbonate method was used to determine photosynthetic versus irradiance (PE) characteristics as described by Lewis and Smith (1983) and Kulk et al. (2011). In short, 17 vials with 2 mL radiolabeled culture samples (0.74 MBq total activity ^14^C‐bicarbonate) were incubated for 60 min at 1°C in a photosynthetron consisting of a temperature controlled aluminum block illuminated by a 250 W lamp (HCI‐TT 250W/942 NDL PB power ball; Osram, München, Germany) with irradiance levels ranging from 4 to 1,158 μmol photons · m^−2^ · s^−1^. Time zero activity and total activity were determined for each PE measurement. Radioactivity in all samples was measured by liquid scintillation spectrometry (Tri‐Carb 2000 CA scintillation counter; Packard, MA, USA) using 10 mL Ultima Gold XR scintillation cocktail (PerkinElmer, MA, USA). Data from the PE measurements were normalized to Chl *a* derived from HPLC measurements and fitted to the empirical model described by Platt et al. (1980) using MatLab software (version 8.3; Mathworks) to estimate the maximum carbon fixation rate (P_max_ in μg C · μg Chl *a* ^−1^ · h^−1^), the initial slope of carbon fixation (α in μg C · μg Chl *a* ^−1^ · h^−1^ [μmol photons · m^−2^ · s^−1^]^−1^), the photoacclimation index (Eκ in μmol photons · m^−2^ · s^−1^), and photoinhibition of carbon fixation (β in μg C · μg Chl *a* ^−1^ · h^−1^ [μmol photons · m^−2^ · s^−1^]^−1^). The electron requirement of carbon fixation (Φ_e,C_ in mol e^−^ · mol C^−1^) was calculated using the ETR and carbon fixation measurements normalized to Chl *a*. PE measurements were also normalized to cellular C (data not shown), which yielded similar results in size scaling compared to Chl *a* normalized measurements.

### Statistical analysis

Differences between the irradiance conditions and the diatom species were statistically tested by ANOVA and Tukey HSD post hoc analysis using STATISTICA software (version 13.0; Statsoft, TX, USA). Before analysis, data were tested for normality and homogeneity of variances and log transformed for further statistical analysis when necessary. Differences were considered significant when *P* < 0.05.

To analyze the role of cell size on growth, photophysiology, and photosynthetic rates, size scaling exponents (SSE) were determined according to Peters (1983). To this end, all parameters were log transformed and linear regression analysis was performed using Sigmaplot software (version 11; Systat Software Inc., San Jose, California, United States of America) to determine the SSE and intercept for each parameter on the basis of biovolume. Additional analysis on the basis of S/V ratios showed similar results (data not shown). Regression coefficients were considered significant within 95% confidence intervals.

## Results

### Growth

**Figure 1:**
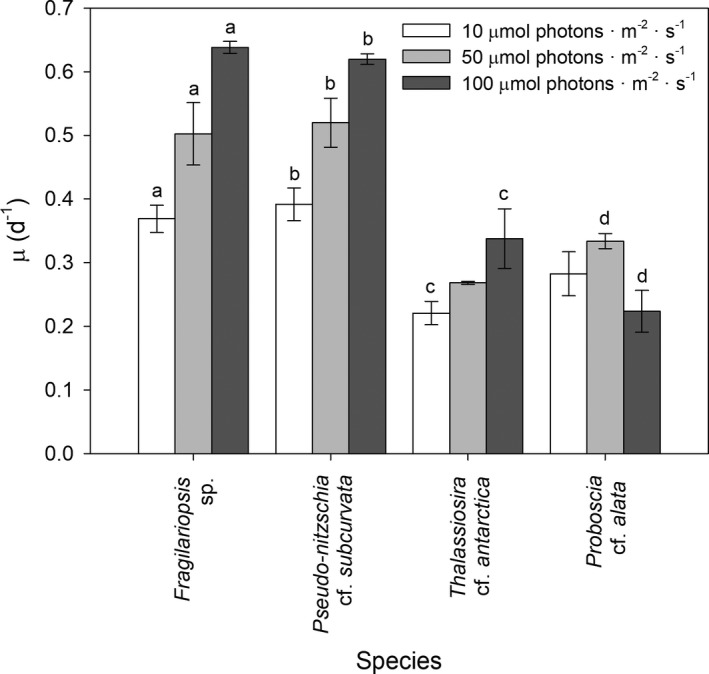
Mean (± SD,* n* = 3) growth rates (μ in · d^−1^) are given for the four Antarctic diatom species *Fragilariopsis* sp. (*Fragilariopsis* sp.), *Pseudo‐nitzschia* cf. *subcurvata* (Ps), *Thalassiosira* cf. *antarctica* (Ta), and *Proboscia* cf. *alata* (Pa) grown at 10, 50, and 100 μmol photons · m^−2^ · s^−1^. Significant differences (*P* < 0.05) between the different irradiance conditions for each diatom species are indicated by identical letters.

Growth rates varied widely among irradiance conditions and diatoms (Fig. 1). Growth rates increased with increasing irradiance in *Fragilariopsis* sp., *Pseudo‐nitzschia subcurvata*, and *Thalassiosira antarctica* (*F*_2,23_ = 32.3, *P* < 0.05). In contrast, in *Proboscia alata*, growth rates increased from 10 to 50 μmol photons · m^−2^ · s^−1^, but decreased at the highest irradiance of 100 μmol photons · m^−2^ · s^−1^ (*F*_2,23_ = 32.3, *P* < 0.001). Highest growth rates were found in the smaller species *Fragilariopsis* sp. and *Pseudo‐nitzschia subcurvata*, ranging from 0.37 ± 0.021 to 0.64 ± 0.009 · d^−1^ and 0.39 ± 0.026 to 0.62 ± 0.008 · d^−1^, respectively (*F*_3,23_ = 129, *P* < 0.05). Lowest growth rates were found in the larger species *Thalassiosira antarctica* and *Proboscia alata* with growth rates ranging from 0.22 ± 0.018 to 0.27 ± 0.002 · d^−1^ and 0.22 ± 0.033 to 0.33 ± 0.012 · d^−1^, respectively (*F*_3,23_ = 129, *P* < 0.05). Calculations of SSE showed a negative relationship between biovolume and growth, which increased with irradiance (Table 1).

**Table 1:** Size scaling exponent and intercept (± SD, *n* = 12) are given for growth (μ), cellular carbon (C), carbon to chlorophyll *a* ratio (C:Chl *a*), cellular chlorophyll *a* (Chl *a*), specific absorption cross section (ā*), maximum quantum yield of PSII (F_v_/F_m_), non‐photochemical quenching (NPQ), slowly relaxing non‐photochemical quenching (NPQ_S_), fast relaxing non‐photochemical quenching (NPQ_F_), maximum electron transport rate (ETR_max_), initial slope of electron transport (α_ETR_), photoacclimation index of electron transport (Eκ_ETR_), maximum carbon fixation rate (P_max_), initial slope of carbon fixation (α), photoacclimation index or carbon fixation (Eκ), photoinhibition of carbon fixation (β), and electron requirement of carbon fixation (Φ_e,C_) at 10, 50 and 100 μmol photons · m^−2^ · s^−1^. Significant regression coefficients within 95% confidence intervals are indicated by * and within 99% confidence intervals by **

	10 μmol photons · m^−2^ · s^−1^			50 μmol photons · m^−2^ · s^−1^			100 μmol photons · m^−2^ · s^−1^		
	Scaling exponent	Intercept	*r* ^2^	Scaling exponent	Intercept	*r* ^2^	Scaling exponent	Intercept	*r* ^2^
μ	−0.070 ± 0.0152	−0.32 ± 0.045	0.679**	−0.092 ± 0.0143	−0.16 ± 0.042	0.806**	−0.142 ± 0.0265	−0.02 ± 0.077	0.742**
C	0.772 ± 0.0202	−0.10 ± 0.060	0.993**	0.822 ± 0.0127	−0.18 ± 0.038	0.998**	0.860 ± 0.0316	−0.35 ± 0.092	0.989**
C:Chl *a*	0.004 ± 0.0241	1.44 ± 0.072	0.003	−0.037 ± 0.0149	1.71 ± 0.044	0.383*	−0.077 ± 0.0155	1.89 ± 0.045	0.755**
Chl *a*	0.767 ± 0.0243	−1.54 ± 0.073	0.990**	0.859 ± 0.0148	−1.89 ± 0.044	0.997**	0.936 ± 0.0387	−2.24 ± 0.112	0.987**
ā*	−0.024 ± 0.0193	−1.65 ± 0.058	0.135	−0.070 ± 0.0248	−1.51 ± 0.073	0.443*	−0.074 ± 0.0232	−1.44 ± 0.067	0.558*
F_v_/F_m_	0.009 ± 0.0052	−0.23 ± 0.016	0.247	0.007 ± 0.0038	−0.26 ± 0.011	0.240	−0.017 ± 0.0083	−0.25 ± 0.024	0.299
NPQ	0.046 ± 0.0178	0.41 ± 0.053	0.399*	−0.013 ± 0.0236	0.71 ± 0.070	0.030	−0.038 ± 0.0292	0.67 ± 0.085	0.177
NPQ_S_	0.137 ± 0.0557	−0.80 ± 0.166	0.377*	0.122 ± 0.0396	−0.76 ± 0.117	0.487*	0.142 ± 0.0756	−1.01 ± 0.219	0.306
NPQ_F_	0.032 ± 0.0159	0.40 ± 0.047	0.285	−0.028 ± 0.0264	0.71 ± 0.078	0.103	−0.054 ± 0.0275	0.67 ± 0.080	0.323
ETR_max_	0.122 ± 0.0345	−1.17 ± 0.103	0.555**	−0.019 ± 0.0301	−0.61 ± 0.089	0.040	−0.032 ± 0.0417	2.57 ± 0.204	0.069
α_ETR_	0.036 ± 0.0365	−2.60 ± 0.109	0.087	−0.075 ± 0.0254	−2.26 ± 0.075	0.467*	−0.063 ± 0.0141	−0.35 ± 0.092	0.714**
Eκ_ETR_	0.086 ± 0.0268	1.43 ± 0.080	0.508**	0.056 ± 0.0258	1.65 ± 0.076	0.320	0.031 ± 0.0408	1.89 ± 0.045	0.068
P_max_	0.021 ± 0.0310	0.23 ± 0.093	0.044	−0.070 ± 0.0394	0.53 ± 0.117	0.240	−0.066 ± 0.0285	0.74 ± 0.085	0.399
α	−0.130 ± 0.0408	−0.76 ± 0.122	0.502**	−0.108 ± 0.0317	−0.85 ± 0.094	0.537**	−0.053 ± 0.0180	−1.04 ± 0.075	0.515*
Eκ	0.150 ± 0.0656	0.99 ± 0.196	0.345*	0.038 ± 0.0539	1.38 ± 0.160	0.047	−0.025 ± 0.0273	1.82 ± 0.079	0.093
β	−0.066 ± 0.0826	−2.90 ± 0.247	0.059	0.087 ± 0.0714	−3.37 ± 0.212	0.129	−0.059 ± 0.0519	−2.69 ± 0.151	0.139
Φ_e,C_	−0.121 ± 0.0310	1.78 ± 0.093	0.602**	−0.010 ± 0.0178	1.22 ± 0.053	0.033	−0.008 ± 0.0270	1.23 ± 0.078	0.011

### Cell size and biovolume

The four Antarctic diatom species varied greatly in cell shape, cell size, biovolume, and S/V ratios. Irradiance had no effect on cell length, biovolume, and S/V ratios in *Fragilariopsis* sp. and *Thalassiosira antarctica* (Table 2). In *Pseudo‐nitzschia subcurvata*, cell length and biovolume increased at higher irradiances (*F*_2,6_ = 13.1, *F*_2,6_ = 12.6, *P* < 0.05), whereas the S/V ratio was highest at 50 μmol photons · m^−2^ · s^−1^ (*F*_2,6_ = 15.8, *P* < 0.01). A contrasting trend was observed in *Proboscia alata*, with a decrease in cell length and biovolume and an increase in the S/V ratio at higher irradiances (*F*_2,6_ = 14.0, *F*_2,6_ = 161, *F*_2,6_ = 1.44, *P* < 0.05). *Fragilariopsis* sp. was the smallest species with significantly shortest (apical) cell length and smallest biovolume, followed by *P. subcurvata*,* T. antarctica,* and *P. alata*, respectively (*F*_2,32_ = 530, *F*_2,32_ = 198, *P* < 0.05; Table 2). Cell length and biovolume between the smallest species *Fragilariopsis* sp. and the largest species *P. alata* varied approximately two orders of magnitude. Related to cell dimensions and biovolume, *Fragilariopsis* sp. showed highest S/V ratios, followed by *Pseudo‐nitzschia subcurvata*,* Proboscia alata,* and *Thalassiosira antarctica*, respectively (*F*_2,32_ = 5,508, *P* < 0.001; Table 2).

**Table 2:** Mean (± SD, *n* = 3) cell length (μm), biovolume (μm^3^) and cell surface to volume ratio (S/V in μm^−1^) are given for the four Antarctic diatom species *Fragilariopsis* sp., *Pseudo‐nitzschia* cf. *subcurvata*,* Thalassiosira* cf. *antarctica*, and *Proboscia* cf. *alata* grown at 10, 50, and 100 μmol photons · m^−2^ · s^−1^. Cell length is given as the (apical) length for *Fragilariopsis* sp. and *P. subcurvata*, as the cell height for *P. alata* and as the diameter for *T. antarctica*. Significant differences (*P* < 0.05) between the different irradiance conditions for each diatom species are indicated by matching letters

	*Fragilariopsis* sp.	*Pseudo‐nitzschia* cf. *subcurvata*	*Thalassiosira* cf. *antarctica*	*Proboscia* cf. *alata*
Cell length				
10	3.56 ± 2.00 × 10^−2^	28.6 ± 2.59^a^	24.2 ± 0.13	271 ± 6.11^c,d^
50	3.50 ± 2.00 × 10^−2^	30.1 ± 1.89^b^	23.8 ± 0.39	220 ± 21.4^c^
100	3.54 ± 6.00 × 10^−2^	36.1 ± 0.73^a,b^	23.4 ± 0.24	220 ± 8.15^d^
Biovolume				
10	14.6 ± 1.58	77 ± 8.48^a^	6,646 ± 57	10,543 ± 93^b^
50	13.9 ± 1.01	93 ± 7.11	6,657 ± 359	8,310 ± 477^b^
100	14.2 ± 0.23	103 ± 0.93^a^	6,288 ± 267	6,360 ± 92^b^
S/V				
10	2.32 ± 9.41 × 10^−2^	1.16 ± 3.31 × 10^−2a^	0.30 ± 6.28 × 10^−4^	0.59 ± 9.01 × 10^−3c^
50	2.36 ± 6.86 × 10^−2^	1.06 ± 1.01 × 10^−2a,b^	0.30 ± 5.58 × 10^−3^	0.60 ± 2.19 × 10^−2d^
100	2.34 ± 8.22 × 10^−3^	1.13 ± 8.89 × 10^−3b^	0.31 ± 5.28 × 10^−3^	0.69 ± 1.10 × 10^−2c,d^

### Cellular carbon

Irradiance had no effect on cellular carbon concentrations in *Fragilariopsis* sp. and *Proboscia alata*, whereas concentrations decreased at 100 μmol photons · m^−2^ · s^−1^ in *Pseudo‐nitzschia subcurvata* (*F*_2,5_ = 8.74, *P* < 0.05) and increased at 50 and 100 μmol photons · m^−2^ · s^−1^ in *Thalassiosira antarctica* (*F*_2,6_ = 30.7, *P* < 0.01; data not shown). Cellular carbon was highly related to cell size and biovolume with lowest concentrations found in the smaller species *Fragilariopsis* sp. (5.32 ± 1.42 pg C · cell^−1^) and highest concentrations found in the larger species *P. alata* (1,129 ± 289 pg C · cell^−1^; data not shown). The positive relationship between biovolume and cellular carbon was also evident in the SSE, at all irradiances (Table 1).

C:Chl *a* increased with irradiance in all diatom species (*F*_2,24_ = 4.09, *P* < 0.05, not significant for *Proboscia alata*; Table 3) and was related to a significant decrease in cellular Chl *a*. When the four diatoms were compared, *Fragilariopsis* sp. and *Pseudo‐nitzschia subcurvata* showed highest C:Chl *a* at higher irradiances, *P. alata* showed highest C:Chl *a* at 10 μmol photons · m^−2^ · s^−1^ (*F*_3,24_ = 4.02, *P* < 0.05) and *Thalassiosira antarctica* showed overall lowest C:Chl *a* (Table 3). The SSE showed a similar trend with a negative relationship between biovolume and C:Chl *a* at 50 and 100 μmol photons · m^−2^ · s^−1^ and no relationship at the lowest irradiance (Table 1).

**Table 3:** Mean (± SD, *n* = 3) cellular chlorophyll *a* (Chl *a* in pg · cell^−1^), carbon to chlorophyll *a* ratio (Chl *a*:C), maximum photosynthetic yield of PSII (F_v_/F_m_), and specific absorption cross section (ā* in m^2^ · mg Chl *a* ^−1^) are given for the four Antarctic diatom species *Fragilariopsis* sp., *Pseudo‐nitzschia* cf. *subcurvata*,* Thalassiosira* cf. *antarctica*, and *Proboscia* cf. *alata* grown at 10, 50, and 100 μmol photons · m^−2^ · s^−1^. Significant differences (*P* < 0.05) between the different irradiance conditions for each diatom species are indicated by identical letters

	*Fragilariopsis* sp.	*Pseudo‐nitzschia* cf. *subcurvata*	*Thalassiosira* cf. *antarctica*	*Proboscia* cf. *alata*
Chl *a*				
10 μmol photons · m^−2^ · s^−1^	0.190 ± 0.032^a,b^	1.027 ± 0.163^c^	28.79 ± 1.501	29.41 ± 5.801^d^
50 μmol photons · m^−2^ · s^−1^	0.121 ± 0.016^a^	0.648 ± 0.064^c^	28.03 ± 0.968	26.41 ± 3.603
100 μmol photons · m^−2^ · s^−1^	0.088 ± 0.030^b^	0.284 ± 0.046^c^	24.06 ± 3.287	19.94 ± 1.136^d^
C:Chl *a*				
10	30.1 ± 2.39^a^	26.3 ± 1.45^b,c^	21.8 ± 1.22^d,e^	38.2 ± 5.37
50	44.7 ± 3.72^a^	46.9 ± 2.77^b^	32.1 ± 1.24^d^	41.9 ± 6.34
100	61.5 ± 4.87^a^	57.0 ± 8.03^c^	35.5 ± 4.09^e^	45.3 ± 1.50
F_v_/F_m_				
10	0.600 ± 2.09 × 10^−3a^	0.618 ± 8.33 × 10^−3b^	0.685 ± 1.67 × 10^−2c^	0.606 ± 8.77 × 10^−3d^
50	0.554 ± 1.31 × 10^−3a^	0.562 ± 1.97 × 10^−3b^	0.606 ± 1.05 × 10^−2c^	0.554 ± 2.43 × 10^−3d^
100	0.518 ± 2.68 × 10^−2a^	0.535 ± 3.60 × 10^−3b^	0.519 ± 7.99 × 10^−3c^	0.437 ± 6.87 × 10^−3d^
ā*				
10	0.020 ± 1.73 × 10^−3a^	0.022 ± 7.89 × 10^−4^	0.014 ± 1.43 × 10^−3^	0.022 ± 6.36 × 10^−4^
50	0.025 ± 5.45 × 10^−3^	0.025 ± 1.31 × 10^−3^	0.013 ± 1.48 × 10^−3^	0.021 ± 4.18 × 10^−3^
100	0.030 ± 5.85 × 10^−3a^	0.026 ± 2.30 × 10^−3^	0.016 ± 1.13 × 10^−3^	0.025 ± 2.74 × 10^−4^

### Pigment composition

**Figure 2:**
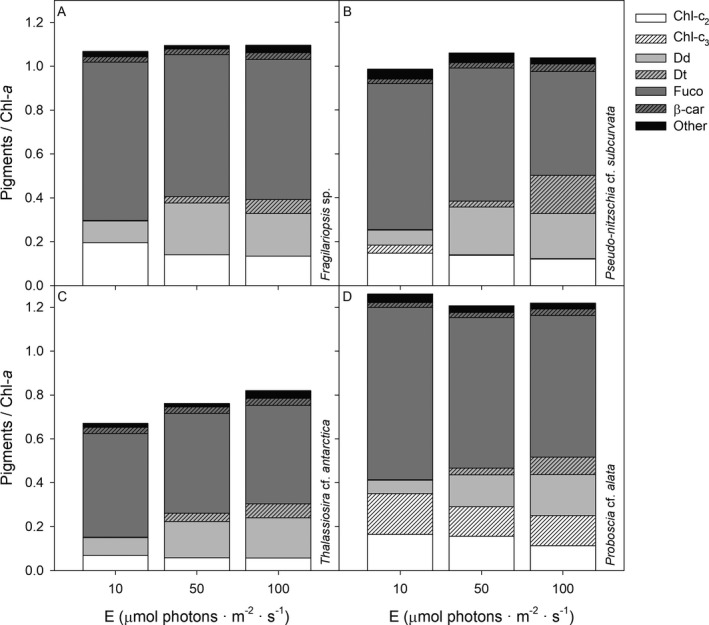
Mean (*n* = 3) pigment compositions are given for the four Antarctic diatom species (A) *Fragilariopsis* sp., (B) *Pseudo‐nitzschia* cf. *subcurvata*, (C) *Thalassiosira* cf. *antarctica*, and (D) *Proboscia* cf. *alata* grown at 10, 50, and 100 μmol photons · m^−2^ · s^−1^. Identified pigments (given as ratio per chlorophyll *a* (Chl *a*)) included chlorophyll *c*_*2*_ (Chl‐c_2_), chlorophyll *c*_*3*_ (Chl‐c_3_), diadinoxanthin (Dd), diatoxanthin (Dt), fucoxanthin (fuco), β‐carotene (β‐car) and other identified pigments (<2.5% of total).

The major pigments chlorophyll *a*, chlorophyll *c*_2_, diadinoxanthin, diatoxanthin, fucoxanthin, and β‐carotene were present in all four Antarctic diatoms species (Fig. 2, Table 3). The pigment composition of *Pseudo‐nitzschia subcurvata* and *Proboscia alata* was further characterized by the presence of chlorophyll *c*_3_ (Fig. 2). Cellular Chl *a* concentrations significantly decreased with increasing irradiance in *Fragilariopsis* sp., *P. subcurvata* and *P. alata* (*F*_2,22_ = 6.44, *P* < 0.05), whereas no changes were observed in *Thalassiosira antarctica* (Table 3). Cellular Chl *a* was highly related to size with lowest concentrations found in *Fragilariopsis* sp. and highest concentrations found in *T. antarctica* and *P. alata* (Table 3). Calculations of SSE also showed a positive relationship between biovolume and Chl *a*, which increased with irradiance (Table 1).

**Figure 3:**
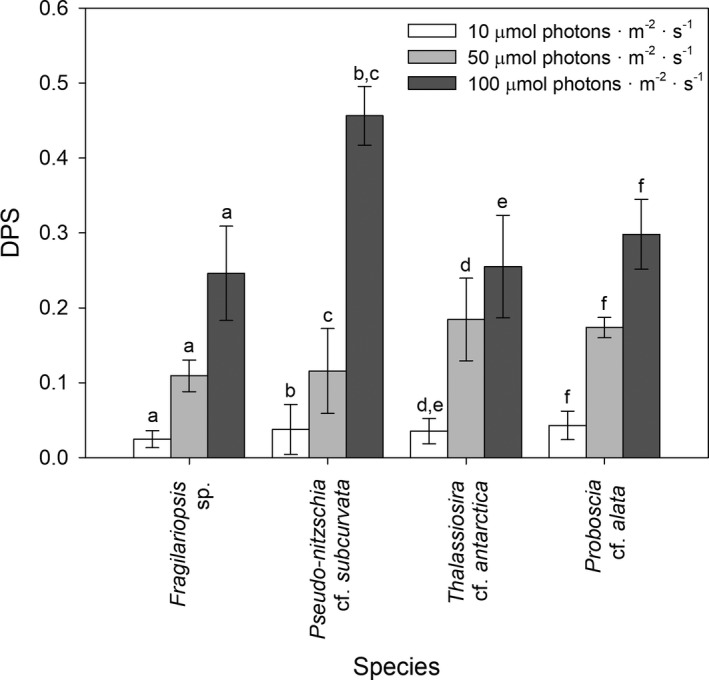
Mean (± SD,* n* = 3) de‐epoxidation state (DPS) of the xanthophyll pigment cycle is given for the four Antarctic diatom species *Fragilariopsis* sp. (*Fragilariopsis* sp.), *Pseudo‐nitzschia* cf. *subcurvata* (Ps), *Thalassiosira* cf. *antarctica* (Ta), and *Proboscia* cf. *alata* (Pa) grown at 10, 50, and 100 μmol photons · m^−2^ · s^−1^. Significant differences (*P* < 0.05) between the different irradiance conditions for each diatom species are indicated by identical letters.

The concentration and activity of xanthophyll pigments showed a strong, uniform response to increasing irradiances in all species (Figs. 2 and 3). Both cellular Dd and Dd/Chl *a* increased from 10 to 50 μmol photons · m^−2^ · s^−1^ (*F*_2,22_ = 141, *P* < 0.05), but did not increase further at 100 μmol photons · m^−2^ · s^−1^. Cellular Dt and Dt/Chl *a* significantly increased at higher irradiances with highest concentrations found at 100 μmol photons · m^−2^ · s^−1^ (*F*_2,22_ = 148, *P* < 0.05). In addition, the DPS of the xanthophyll pigment cycle significantly increased at higher irradiances, with the strongest increase observed in *Pseudo‐nitzschia subcurvata* (Fig. 3). No differences in xanthophyll pigment ratios were observed between the four diatoms and *Fragilariopsis* sp., *Pseudo‐nitzschia subcurvata*,* Thalassiosira antarctica,* and *Proboscia alata* showed similar Dd/Chl *a* and Dt/Chl *a* (Fig. 2). Cellular concentrations of the xanthophyll pigments had similar SSE as Chl *a*, whereas no relationship with biovolume was observed for Dd/Chl *a*, Dt/Chl *a* and/or the DPS of the xanthophyll pigment cycle (data not shown).

Irradiance also affected the concentrations of other accessory pigments, with a decrease in Chl *c*_*2*_/*a* and Chl *c*_*3*_/*a* at higher irradiances (*F*_2,22_ = 46.6, *F*_2,22_ = 78.7, *P* < 0.05) in all species. Moreover, Fuco/Chl *a* decreased at higher irradiances in *Pseudo‐nitzschia subcurvata* and *Proboscia alata* (*F*_2,22_ = 94.3, *P* < 0.05), but was not affected by irradiance in *Fragilariopsis* sp. and *Thalassiosira antarctica* (Fig. 2). When the four diatoms were compared, Chl *c*_*2*_/*a* and Fuco/Chl *a* were higher in *Fragilariopsis* sp., *P. subcurvata* and *P. alata* (ranging between 0.113–0.196 and 0.473–0.785, respectively) compared to *T. antarctica* (ranging from 0.057–0.069 and 0.449–0.472, respectively; *F*_3,22_ = 180, *F*_3,22_ = 298, *P* < 0.05; Fig. 2). In addition, Chl *c*_*2*_/*a* was lower in *P. subcurvata* (0.002–0.037) compared to *P. alata* (0.137–0.186; *F*_3,22_ = 1,505, *P* < 0.001; Fig. 2). The SSE of cellular concentrations of the accessory pigments were similar to those of Chl *a*, whereas no relationship with biovolume was observed for ratios per Chl *a* (data not shown).

### Absorption spectra

**Figure 4:**
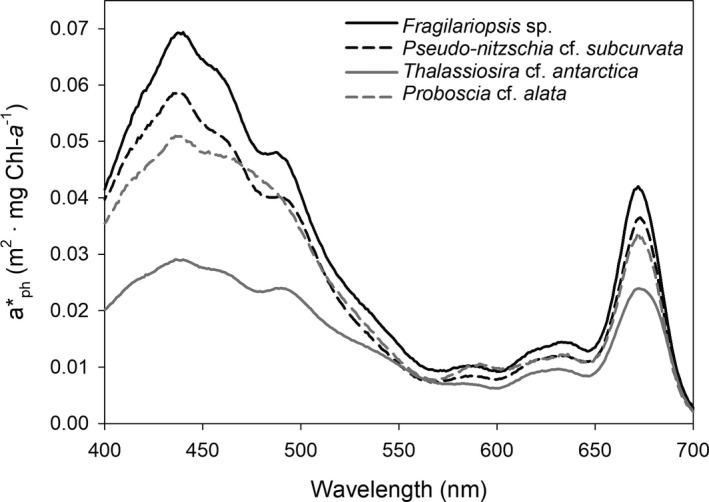
Mean (*n* = 3) specific absorption coefficient a*_ph_(λ) is given for *Fragilariopsis* sp. (*Fragilariopsis* sp.), *Pseudo‐nitzschia* cf. *subcurvata* (Ps), *Thalassiosira* cf. *antarctica* (Ta), and *Proboscia* cf. *alata* (Pa) grown at 100 μmol photons · m^−2^ · s^−1^.

Absorption characteristics varied among irradiance conditions and diatoms. In *Fragilariopsis* sp., ā* increased with increasing irradiances (*F*_2,22_ = 5.97, *P* < 0.05) and a similar, but not significant trend was observed in the other species (Table 3). ā* was lower in *Thalassiosira antarctica* compared to the other Antarctic diatom species (*F*_3,22_ = 23.5, *P* < 0.05), whereas *Fragilariopsis* sp., *Pseudo‐nitzschia subcurvata,* and *Proboscia alata* showed similar absorption characteristics (Fig. 4, Table 3). The SSE of ā* showed a negative relationship with biovolume at 50 and 100 μmol photons · m^−2^ · s^−1^, whereas no relationship with biovolume was observed at 10 μmol photons · m^−2^ · s^−1^ (Table 1).

### PSII chlorophyll fluorescence characteristics

F_v_/F_m_ decreased with increasing irradiances in all diatom species (*F*_2,22_ = 407, *P* < 0.05), with the strongest decrease (between 24% and 28%) observed in the larger species *Thalassiosira antarctica* and *Proboscia alata* (Table 3). Highest F_v_/F_m_ was observed in *T. antarctica* (*F*_3,22_ = 68.3, *P* < 0.01) at 10 and 50 μmol photons · m^−2^ · s^−1^, followed by *Pseudo‐nitzschia subcurvata*,* P. alata,* and *Fragilariopsis* sp., respectively, whereas F_v_/F_m_ was highest in *P. subcurvata* at 100 μmol photons · m^−2^ · s^−1^ (*F*_3,22_ = 68.3, *P* < 0.05; Table 3). The SSE showed no clear relationship between biovolume and F_v_/F_m_ (Table 1).

**Figure 5:**
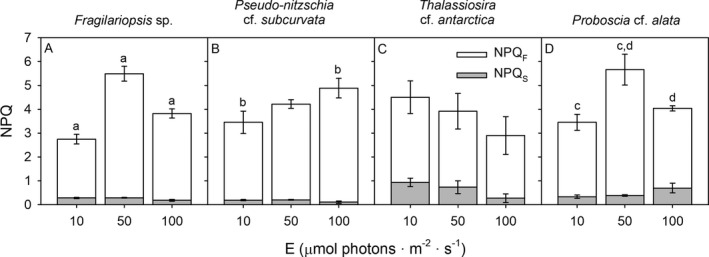
Mean (± SD,* n* = 3) non‐photochemical quenching (NPQ) is given for the four Antarctic diatom species (A) *Fragilariopsis* sp., (B) *Pseudo‐nitzschia* cf. *subcurvata*, (C) *Thalassiosira* cf. *antarctica*, and (D) *Proboscia* cf. *alata* grown at 10, 50, and 100 μmol photons · m^−2^ · s^−1^. The contribution of fast relaxing non‐photochemical quenching (NPQ_F_) to total NPQ is indicated by light gray and the contribution of slowly relaxing non‐photochemical quenching (NPQ_S_) to total NPQ is indicated by white. Significant differences (*P* < 0.05) in total NPQ between the different irradiance conditions for each diatom species are indicated by identical letters.

All diatom species showed high levels of NPQ upon short term high irradiance exposure (Fig. 5). In general, the contribution of NPQ_S_ to total NPQ was low and a large fraction of NPQ (77%–98%) was related to NPQ_F_. Acclimation to the different irradiances affected NPQ in various ways. In *Fragilariopsis* sp., highest levels of total NPQ and NPQ_F_ were found at 50 μmol photons · m^−2^ · s^−1^, followed by 100 and 10 μmol photons · m^−2^ · s^−1^, respectively (*F*_2,22_ = 15.9, *F*_2,22_ = 22.1, *P* < 0.01), whereas NPQ_S_ decreased at higher irradiances (*F*_2,22_ = 8.84, *P* < 0.001). In *Pseudo‐nitzschia subcurvata*, total NPQ and NPQ_F_ increased, whereas NPQ_S_ decreased at higher irradiances (*F*_2,22_ = 15.9, *F*_2.22_ = 22.1, *F*_2,22_ = 8.84, *P* < 0.05). In *Thalassiosira antarctica*, total NPQ showed a decreasing trend with increasing irradiance (not significant), whereas NPQ_S_ decreased at higher irradiances (*F*_2,22_ = 8.84, *P* < 0.05) and NPQ_F_ remained unaffected by irradiance. And in *Proboscia alata*, highest levels of total NPQ and NPQ_F_ were found at 50 μmol photons · m^−2^ · s^−1^ compared to 10 and 100 μmol photons · m^−2^ · s^−1^ (*F*_2,22_ = 15.9, *F*_2,22_ = 22.1, *P* < 0.05) and NPQ_S_ increased at higher irradiance (*F*_2,22_ = 8.84, *P* < 0.05). When the different diatoms were compared, highest levels of NPQ were found in *Fragilariopsis* sp. and *P. alata* at 50 μmol photons · m^−2^ · s^−1^ (*F*_3,22_ = 1.80, *P* < 0.05; Fig. 5). Highest levels of NPQ_S_ were found in *T. antarctica*, whereas the lowest levels were found in *P. subcurvata* at 100 μmol photons · m^−2^ · s^−1^ (*F*_3,22_ = 29.0, *P* < 0.05). Highest values of NPQ_F_ were found in *Fragilariopsis* sp., *P. subcurvata* and *P. alata* at 50, 100, and 50 μmol photons · m^−2^ · s^−1^, respectively (*F*_3,22_ = 5.49, *P* < 0.05) and the four species showed similar levels of NPQ_F_ under all other conditions. The SSE showed a positive relationship between biovolume and NPQ and NPQs at lower irradiances, whereas no significant relationship with biovolume was observed for NPQ_F_ (Table 1).

### Electron transport rates

ETR_max_ increased at higher irradiances in *Fragilariopsis* sp. (*F*_2,22_ = 8.18, *P* < 0.01) and *Thalassiosira antarctica* (not significant; Table 4). In contrast, highest ETR_max_ was found at 50 μmol photons · m^−2^ · s^−1^ in both *Pseudo‐nitzschia subcurvata* (*F*_2,22_ = 8.18, *P* < 0.05) and *Proboscia alata* (not significant). When the different diatoms were compared, ETR_max_ was highest in *T. antarctica* and *Fragilariopsis* sp. at 100 μmol photons · m^−2^ · s^−1^ (*F*_3,22_ = 3.27, *P* < 0.05) and lowest in *Fragilariopsis* sp. at 10 μmol photons · m^−2^ · s^−1^ (*F*_3,22_ = 3.27, *P* < 0.05). The SSE showed a relationship between biovolume and ETR_max_ at 10 μmol photons · m^−2^ · s^−1^, but not at higher irradiances (Table 1). α_ETR_ increased at higher irradiances in the smaller species *Fragilariopsis* sp. and *P. subcurvata* (*F*_2,22_ = 3.58, *P* < 0.05), but remained unaffected by irradiance in the larger species *T. antarctica* and *P. alata* (Table 4). α_ETR_ was not significantly different between the four diatoms, but showed a decreasing trend with size at higher irradiances, as was also evident from the SSE (Tables 1 and 4). Eκ_ETR_ showed an increasing trend with irradiance in *Fragilariopsis* sp. and *T. antarctica*, remained unaffected by irradiance in *P. subcurvata* and was highest at 50 μmol photons · m^−2^ · s^−1^ in *P. alata* (Table 4). *Thalassiosira antarctica* acclimated to higher irradiances compared to the other three diatom species (*F*_3,22_ = 10.3, *P* < 0.05), whereas Eκ_ETR_ was similar between *Fragilariopsis* sp., *Pseudo‐nitzschia subcurvata,* and *Proboscia alata* (except for *Fragilariopsis* sp. at 10 μmol photons · m^−2^ · s^−1^). The SSE showed a positive relationship between biovolume and Eκ_ETR_ at 10 μmol photons · m^−2^ · s^−1^ (Table 1).

**Table 4:** Mean ( ±  SD, *n* = 3) maximum electron transport rate (ETR_max_ in mol e^−^ · μg Chl *a* ^−1^ · h^−1^), initial slope of electron transport (α_ETR_ in mol e^−^ · μg Chl *a* ^−1^ · h^−1^ (μmol photons · m^−2^ · s^−1^)^−1^), photoacclimation index for electron transport (Eκ_ETR_ in μmol photons · m^−2^ · s^−1^), maximum rate of carbon fixation (P_max_ in μg C · μg Chl *a* ^−1^ · h^−1^), initial slope of carbon fixation (α in μg C · μg Chl *a* ^−1^ · h^−1^ (μmol photons · m^−2^ · s^−1^)^−1^), photoacclimation index for carbon fixation (Eκ in μmol photons · m^−2^ · s^−1^), and photoihbition of carbon fixation (β in μg C · μg Chl *a* ^−1^ · h^−1^ (μmol photons · m^−2^ · s^−1^)^−1^) are given for the four Antarctic diatom species *Fragilariopsis* sp., *Pseudo‐nitzschia* cf. *subcurvata*,* Thalassiosira* cf. *antarctica*, and *Proboscia* cf. *alata* grown at 10, 50, and 100 μmol photons · m^−2^ · s^−1^. Significant differences (*P* < 0.05) between the different irradiance conditions for each diatom species are indicated by identical letters

	*Fragilariopsis* sp.	*Pseudo‐nitzschia* cf. *subcurvata*	*Thalassiosira* cf. *antarctica*	*Proboscia* cf. *alata*
ETR_max_				
10 μmol photons · m^−2^ · s^−1^	0.068 ± 0.025^a^	0.131 ± 0.023^b,c^	0.249 ± 0.084	0.166 ± 0.003
50 μmol photons · m^−2^ · s^−1^	0.173 ± 0.011^a^	0.254 ± 0.033^b^	0.219 ± 0.081	0.206 ± 0.059
100 μmol photons · m^−2^ · s^−1^	0.307 ± 0.007^a^	0.206 ± 0.003^c^	0.328 ± 0.106	0.151 ± 0.011
α_ETR_				
10	2.15 × 10^−3^ ± 6.56 × 10^−4a^	2.74 × 10^−3^ ± 6.02 × 10^−4b,c^	3.62 × 10^−3^ ± 1.43 × 10^−3^	3.57 × 10^−3^ ± 6.25 × 10^−4^
50	4.20 × 10^−3^ ± 8.19 × 10^−4^	4.53 × 10^−3^ ± 3.56 × 10^−4b^	2.83 × 10^−3^ ± 9.03 × 10^−4^	2.92 × 10^−3^ ± 9.11 × 10^−4^
100	5.55 × 10^−3^ ± 1.19 × 10^−3a^	4.51 × 10^−3^ ± 9.36 × 10^−5c^	3.63 × 10^−3^ ± 3.67 × 10^−4^	3.74 × 10^−3^ ± 3.14 × 10^−4^
Eκ_ETR_				
10	31.5 ± 1.95	48.3 ± 6.56	70.0 ± 4.72	47.8 ± 10.32
50	45.0 ± 14.60	56.0 ± 4.41	76.4 ± 3.69	74.4 ± 26.86
100	56.7 ± 10.11	45.7 ± 1.63	88.9 ± 21.14	40.5 ± 0.60
P_max_				
10	1.39 ± 0.179^a^	2.64 ± 0.149^b,c^	2.03 ± 0.164^d^	1.99 ± 0.765
50	2.86 ± 1.075	3.65 ± 0.436^b^	1.62 ± 0.136^e^	1.80 ± 0.380
100	4.95 ± 1.174^a^	3.65 ± 0.026^c^	3.47 ± 1.316^d,e^	2.93 ± 0.362
α				
10	0.179 ± 5.12 × 10^−2^	0.062 ± 2.77 × 10^−3^	0.054 ± 1.65 × 10^−2^	0.063 ± 1.05 × 10^−2^
50	0.125 ± 4.43 × 10^−2^	0.071 ± 6.44 × 10^−3^	0.062 ± 2.18 × 10^−2^	0.053 ± 1.43 × 10^−2^
100	0.079 ± 1.69 × 10^−2^	0.075 ± 1.14 × 10^−2^	0.062 ± 1.30 × 10^−3^	0.051 ± 7.32 × 10^−3^
Eκ				
10	8.5 ± 3.89^a^	42.5 ± 4.21	41.2 ± 16.29	31.2 ± 8.72
50	22.0 ± 1.84^b^	51.7 ± 8.74	28.7 ± 11.80	36.5 ± 13.26
100	68.0 ± 9.12^a,b^	49.3 ± 7.16	56.0 ± 21.62	57.0 ± 1.07
β				
10	6.80 × 10^−4^ ± 2.34 × 10^−4a^	1.22 × 10^−3^ ± 3.18 × 10^−4^	5.50 × 10^−4^ ± 3.61 × 10^−5^	3.77 × 10^−4^ ± 2.62 × 10^−4c^
50	7.75 × 10^−4^ ± 2.12 × 10^−5b^	8.03 × 10^−4^ ± 2.90 × 10^−4^	8.27 × 10^−4^ ± 3.86 × 10^−4^	6.07 × 10^−4^ ± 2.64 × 10^−4d^
100	1.32 × 10^−3^ ± 4.58 × 10^−5a,b^	7.55 × 10^−4^ ± 1.48 × 10^−4^	9.40 × 10^−4^ ± 2.52 × 10^−4^	1.05 × 10^−3^ ± 5.66 × 10^−5c,d^

### Carbon fixation rates

P_max_ increased at higher irradiances in all species (*F*_2,21_ = 26.8, *P* < 0.05, except for *Proboscia alata*) with the strongest increase (356%) observed in *Fragilariopsis* sp. (Table 4). Irradiance affected the comparison between the different diatoms. At the lowest irradiance, P_max_ was lowest in *Fragilariopsis* sp. and highest in *Pseudo‐nitzschia subcurvata* (*F*_3,21_ = 7.88, *P* < 0.05). At 50 μmol photons · m^−2^ · s^−1^, P_max_ was higher in the smaller species *Fragilariopsis* sp. and *P. subcurvata* compared to the larger species *Thalassiosira antarctica* and *P. alata* (*F*_3,21_ = 7.88, *P* < 0.05). And at the highest irradiance, *Fragilariopsis* sp. showed highest P_max_ (*F*_3,21_ = 7.88, *P* < 0.01), whereas P_max_ was similar for the other three species (Table 4). Overall, *Fragilariopsis* sp. showed both lowest and highest P_max_ at 10 and 100 μmol photons · m^−2^ · s^−1^, respectively (*F*_3,21_ = 7.88, *P* < 0.05) and no clear trend with biovolume was observed (Table 1). α showed a decreasing trend with irradiance in *Fragilariopsis* sp., whereas α remained unaffected by irradiance in the other three Antarctic diatom species (Table 4). *Fragilariopsis* sp. showed highest α at 10 and 50 μmol photons · m^−2^ · s^−1^ (*F*_3,21_ = 23.2 *P* < 0.01), whereas α was similar in *P. subcurvata*,* T. antarctica* and *P. alata*. In addition, the SSE showed a negative relationship between biovolume and α (Table 1). Eκ increased significantly with irradiance in *Fragilariopsis* sp. (*F*_2,21_ = 18, *P* < 0.01) and a similar trend was observed in *T. antarctica* and *P. alata* (not significant), whereas Eκ was unaffected by irradiance in *P. subcurvata* (Table 4). Related to the strong increase in Eκ with irradiance, *Fragilariopsis* sp. showed both lowest and highest Eκ at 10 and 100 μmol photons · m^−2^ · s^−1^, respectively, compared to the other Antarctic diatom species (*F*_3,21_ = 2.44, *P* < 0.05). A positive relationship between biovolume and Eκ was observed at 10 μmol photons · m^−2^ · s^−1^, but not at higher irradiances (Table 1). β increased with irradiance in *Fragilariopsis* sp., *T. antarctica* and *P. alata* (*F*_2,21_ = 3.57, *P* < 0.05, not significant for *T. antarctica*), whereas β showed a decreasing trend at higher irradiances in *P. subcurvata* (not significant; Table 4). β was similar among the four Antarctic diatom species at 50 μmol photons · m^−2^ · s^−1^, whereas *P. subcurvata* and *Fragilariopsis* sp. showed highest β at 10 and 100 μmol photons · m^−2^ · s^−1^, respectively (*F*_3,21_ = 2.37, *P* < 0.05). No clear relationship with biovolume was observed for β (Table 1).

### Electron requirement of carbon fixation

**Figure 6:**
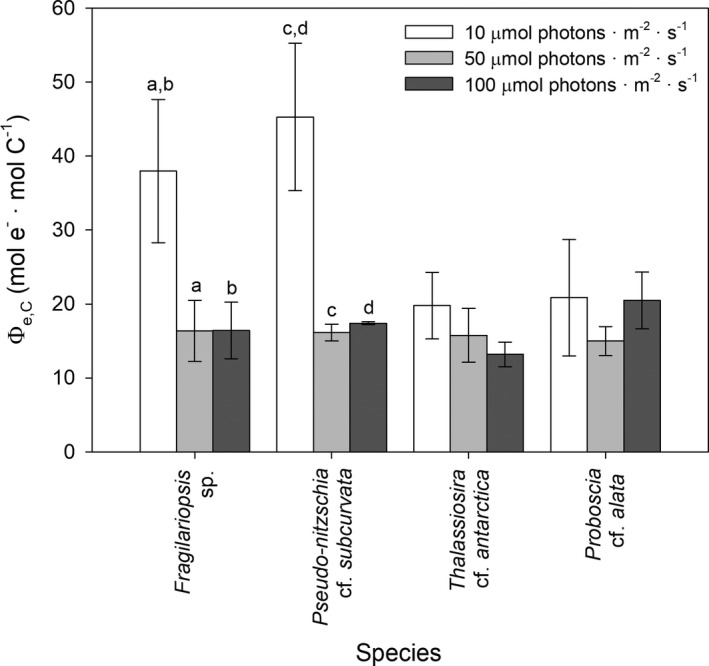
Mean (± SD,* n* = 3) electron requirement for carbon fixation (Φ_e,C_ in mol e^−^ · mol C^−1^) are given for the four Antarctic diatom species *Fragilariopsis* sp. (*Fragilariopsis* sp.), *Pseudo‐nitzschia* cf. *subcurvata* (Ps), *Thalassiosira* cf. *antarctica* (Ta), and *Proboscia* cf. *alata* (Pa) grown at 10, 50, and 100 μmol photons · m^−2^ · s^−1^. Significant differences (*P* < 0.05) between the different irradiance conditions for each diatom species are indicated by identical letters.

In all species, Φ_e,C_ was higher than the theoretical value of 4–6 mol e^−^ · mol C^−1^ and ranged from 13.2 ± 1.67 to 45.3 ± 1.14 mol e^−^ · mol C^−1^ (Fig. 6). Φ_e,C_ was significantly higher at 10 compared to 50 and 100 μmol photons · m^−2^ · s^−1^ in *Fragilariopsis* sp. and *Pseudo‐nitzschia subcurvata* (*F*_2,22_ = 28.2, *P* < 0.05), whereas Φ_e,C_ was similar among the different irradiances in *Thalassiosira antarctica* and *Proboscia alata*. The smaller species *Fragilariopsis* sp. and *Pseudo‐nitzschia subcurvata* showed significantly higher Φ_e,C_ compared to the larger species *T. antarctica* and *P. alata* at the lowest irradiance of 10 μmol photons · m^−2^ · s^−1^ (*F*_2,22_ = 6.92, *P* < 0.05), but Φ_e,C_ was similar between the different diatoms at higher irradiances. A similar trend was observed in the SSE (Table 1).

## Discussion

In northern Marguerite Bay, the onset of the phytoplankton bloom and the distribution of specific diatom species are related to water column stability and the consequent irradiance climate phytoplankton experience (Clarke et al. 2008, Venables et al. 2013). Despite the observation of different cell size classes throughout the phytoplankton growth season, little is known about the role of size‐dependent photophysiological responses in bloom formation and succession of Antarctic diatoms. Strong size related differences in growth rate were observed in this study, with the smaller species *Fragilariopsis* sp. and *Pseudo‐nitzschia subcurvata* showing much higher growth rates compared to the larger species *Thalassiosira antarctica* and *Proboscia alata*, especially at higher irradiances. Growth rates of *Fragilariopsis* sp. and *P. subcurvata* were within the range reported for other small Antarctic diatom species (Boelen et al. 2011, Trimborn et al. 2013, Zhu et al. 2016), whereas growth rates in *T. antarctica* and *P. alata* were somewhat lower compared to earlier reports for larger Antarctic diatoms species (Timmermans et al. 2001a, Heiden et al. 2017). The negative relationship between cell size and growth has previously been reported for temperate diatoms and other phytoplankton species and has been associated with variations in light harvesting capacity and photoinhibition at high and/or excessive irradiances (Agustí 1991, Finkel 2001, Key et al. 2010).

In this study, *Fragilariopsis* sp., *Pseudo‐nitzschia subcurvata,* and *Proboscia alata* showed very similar photophysiology and photoacclimation in response to increasing irradiances. These three Antarctic diatom species showed a reduction in cellular chlorophyll *a* concentrations and subsequent changes in the carbon to chlorophyll *a* ratio upon acclimation to higher irradiances, which has been observed in many other phytoplankton species from both temperate and polar regions (Falkowski and LaRoche 1991, Kropuenske et al. 2009, Arrigo et al. 2010). The decrease in cellular chlorophyll *a* in *Fragilariopsis* sp., *P. subcurvata,* and *P. alata* was associated with an increase in chlorophyll *a* specific absorption. *Thalassiosira antarctica* deviated from these trends with similar concentrations of cellular chlorophyll *a* and chlorophyll *a* specific absorption under various irradiance conditions. Despite the contrasting observations in *T. antarctica*, both cellular chlorophyll *a* and absorption per chlorophyll *a* were related to cell size and biovolume. Earlier research in Antarctic diatoms species suggested that smaller species thrive under lower irradiance conditions compared to larger species (Karentz et al. 1991, Timmermans et al. 2001a,b). This study showed that this is related to relative high pigment concentrations, high pigment absorption, and high ETR in smaller Antarctic diatoms, which has also been observed in temperate centric diatoms (Finkel 2001, Key et al. 2010). Moreover, the relationship between size and the light harvesting processes became stronger at higher irradiances, confirming earlier theoretical based observations that photoacclimation affects the size scaling of photophysiology and growth (Mei et al. 2009).

In contrast to light harvesting processes, photoprotection and photoinhibition showed no clear relationship with cell size and/or biovolume. The concentration and activity of the xanthophyll pigment cycle was uniform among the four different Antarctic diatom species, with significantly higher xanthophyll cycle pigment concentrations and activity at higher irradiances. This has previously been observed in other Antarctic diatom species such as *Chaetoceros brevis* and *Fragilariopsis cylindrus* (Kropuenske et al. 2009, Van de Poll et al. 2009, Arrigo et al. 2010, Boelen et al. 2011). In addition, high levels of NPQ were found in the studied diatom species, which were higher compared to those previously reported for other Antarctic phytoplankton species (Alderkamp et al. 2012, Trimborn et al. 2013, Hoppe et al. 2015), but similar to the Antarctic diatom *C. brevis* and natural phytoplankton communities (Van De Poll et al. 2011). In this study, levels of NPQ_F_ were not directly related to the de‐epoxidation of the xanthophyll pigment cycle. It has earlier been suggested that NPQ can be underestimated in Antarctic diatoms at higher irradiances due to a persistent proton gradient across the thylakoid membrane and the consequent slow epoxidation of the xanthophyll pigment cycle (Goss et al. 2006, Kropuenske et al. 2009). The high levels of photoprotection resulted in relatively low photoinhibition in the four Antarctic diatom species, as has previously been reported for the Antarctic diatom species *Fragilariopsis* sp. in comparison to *Phaeocystis antarctica* (Kropuenske et al. 2009, 2010). In contrast to earlier observations in temperate diatoms, the smaller Antarctic diatoms in this study did not show increased susceptibility to photoinhibition at high and/or excessive irradiances (Finkel 2001, Key et al. 2010). This suggests that photoinhibition played a limited role in size scaling of growth in Antarctic diatoms and that photoprotection is sufficient in both small and large Antarctic diatoms to acclimate to a variety of irradiance conditions.

Photosynthetic rates in the studied diatom species were within the range earlier reported for Antarctic diatoms and other phytoplankton species (Mills et al. 2010, Alderkamp et al. 2012, Hoppe et al. 2015), but were highly variable with cell size and biovolume and between irradiance conditions. Moreover, the observed electron transport and carbon fixation rates could not explain the success in growth of the smaller species *Fragilariopsis* sp. and *Pseudo‐nitzschia subcurvata*, especially at the lowest and highest irradiance conditions. This suggests that photosynthetic processes downstream of PSII play an important role in the growth of the Antarctic diatoms. In this study, the relatively high electron requirement for carbon fixation of the small diatom species (versus the theoretical 4–6 mol e^−^ · mol C^−1^; Genty et al. 1989, Sugget et al. 2009) indicated that energy is lost during the process of photosynthesis. Relatively high electron requirements have been reported in various phytoplankton species and natural phytoplankton communities (Sugget et al. 2009, Lawrenz et al. 2013) and have been associated with increased dissipation of excess energy as heat by the xanthophyll pigment cycle and/or alternative electron transport pathways (Olaizola et al. 1994, Prášil et al. 1996, Raven 2011). Although the observed patterns in photoprotection could explain the overall high electron requirements for carbon fixation in all studied species, there was no size scaling observed in photoprotection. Possibly, alternative electron transport pathways, such as connectivity between PSII reaction centers and/or the Mehler reaction play a role in the size scaling of growth as species specific differences have been observed in various Antarctic diatoms (Trimborn et al. 2014, Heiden et al. 2017). Alternatively, carbon fixation can be limited by the activity of RuBisCO, especially at lower temperatures (Young et al. 2014). Antarctic diatoms and natural Antarctic phytoplankton communities generally have much higher cellular concentrations of RuBisCO, as well as a higher carboxylation and turnover rates at lower temperatures (Young et al. 2014). Despite these adaptations, the relatively low turnover rates of RuBisCO at low temperatures suggests that diatoms in the WAP region may be fixing carbon near their theoretical maximum rate (Young et al. 2014). Cell size may play an additional role, with smaller diatom species showing higher carbon fixation rates per RuBisCO, but larger diatom species investing in higher cellular concentrations of RuBisCO (Wu et al. 2014). Moreover, the activity and gene expression of RuBisCO decreases at lower irradiances in Antarctic diatoms (Falkowski and La Roche 1991, MacIntyre et al. 1996, Boelen et al. 2011), potentially explaining the overproduction of electrons relative to carbon fixation in *Fragilariopsis* sp. and *Pseudo‐nitzschia subcurvata* at lower irradiances.

In this study, the smaller diatoms *Fragilariopsis* sp. and *P. subcurvata* showed highest growth rates, indicating that these species would be able to outcompete larger diatoms such as *Thalassiosira antarctica* and *Proboscia alata* in natural phytoplankton communities. In northern Marguerite Bay, the species composition of the phytoplankton community changes throughout the season, with smaller diatoms such as *Chaetoceros* and *Fragilariopsis* dominating the phytoplankton community during winter and early spring, whereas large centric diatoms such *Odontella*,* Thalassiosira,* and *Proboscia* dominate during the peak phytoplankton bloom (Clarke et al. 2008, Annett et al. 2010, A. Buma, pers. obs.). The observed size‐dependent photophysiology of Antarctic diatoms supports the occurrence of small diatom species earlier in the phytoplankton growth season, with high light harvesting capacity and ETR and low photoinbition in *Fragilariopsis* sp. and *P. subcurvata*. However, the success of larger diatom species during the phytoplankton bloom could not be explained by the variations observed in photophysiology. In addition to irradiance, nutrient availability might play an important role in the bottom‐up control of smaller and larger Antarctic phytoplankton species (Raven 1998, Timmermans et al. 2001a, Finkel et al. 2010). In northern Marguerite Bay, the availability of trace metals is dependent on sea ice melt, glacial melt water input and wind induced mixing, whereas the availability of macronutrients is tightly coupled to phytoplankton growth (Bown et al. 2017). Early in the season, smaller phytoplankton species might benefit from low availability of trace metals due to a relatively high surface‐area‐to‐volume ratio and low nutrient requirement for growth (Raven 1998, Timmermans et al. 2001a, Finkel et al. 2010). Throughout the season, sea ice melt, glacial melt water input and wind induced mixing release more trace elements into the water column (Bown et al. 2017), thereby supporting growth of larger phytoplankton cells. As macronutrients are drawn down during the phytoplankton bloom, larger phytoplankton cells might benefit on short timescales due to a relatively greater storage capacity and potential lower metabolic costs of growth (Finkel et al. 2010, Key et al. 2010, Grover 2011). Variations in top‐down control of small and large phytoplankton species may play an additional role during the phytoplankton bloom in Marguerite Bay. It has been shown that Antarctic phytoplankton communities dominated by smaller phytoplankton species experience higher grazing pressure compared to those dominated by larger phytoplankton species (Storm and Welschmeyer 1991, Garibotti et al. 2003, Smith and Lancelot 2004). Moreover, grazing is dependent on the density of phytoplankton cells in the water column (Landry 1993, Behrenfeld 2010, Garzio and Steinberg 2013). This suggests that low phytoplankton biomass earlier in the season will result in relatively low grazing pressure on both small and large diatoms, whereas during the phytoplankton bloom, grazing of small diatom species increases due to the relatively high abundance of these cells and relatively high grazing pressure, thereby increasing the competitive success of larger diatom species later in the season.

## Conclusions

This study showed that the four Antarctic diatom species *Fragilariopsis* sp., *Pseudo‐nitzschia subcurvata*,* Thalassiosira antarctica*, and *Proboscia alata* are able to acclimate to a variety of irradiance conditions found in coastal areas of the WAP. Although growth rates were related to size, differences in photophysiology and photosynthetic rates could not solely be explained by the two order of magnitude difference in cell size and biovolume of the four Antarctic diatom species. Size related differences in photosynthetic processes associated with light harvesting, as well as the efficiency of light harvesting at lower irradiances showed the potential success of smaller Antarctic diatom species early and late in the phytoplankton growth season. However, the response of the four Antarctic diatom species to various irradiance conditions could not explain the observed patterns in community size structure in northern Marguerite bay during the phytoplankton bloom, indicating that other factors such as nutrient availability and/or grazing pressure might play a more important role in the succession of different diatom species during the phytoplankton growth season in the WAP.

We thank Maria van Leeuwe for analysis of POC and the station and support staff at Rothera Research Station and the British Antarctic Survey who were involved in the summer 2013/2014 field campaign. This work was supported by the Netherlands Organization for Scientific Research (NWO) as part of the Netherlands Polar Programme, grant number 866.14.103 (GK), grant number 866.12.408 (WHP), and grant number 866.10.105 (PDR).
